# Supplementary material for: Are carbon emissions trading and green financial instruments synergistic? -Comprehensive quantitative research based on content analysis
Source: PLoS One. 2024 Mar 7;19(3):e0298601. doi: 10.1371/journal.pone.0298601 (PMC10919601; doi:10.1371/journal.pone.0298601)
Supplement: S1 Data — (ZIP) [file pone.0298601.s001.zip › Data/地级市Z_F工作报告.zip!/╔╜╢1⁄2/┴┘╥╩╩╨/└┤╫╘╣1⁄2╓┌║┼í╛┬φ┐╦╩2╛▌═°í┐_2017.docx]

各位代表：

现在，我代表市人民政府向大会报告工作，请予审议，并请市政协委员和其他列席会议的同志提出意见。

一、过去五年工作回顾二号首长

市十八届人大一次会议以来，在中央、省委省政府和市委的坚强领导下，市政府按照“走在前列”的要求，大力实施“八大战略”，打好“六场硬仗”，全面推进稳增长、促改革、调结构、惠民生、防风险各项工作，全市经济社会保持平稳健康发展，“大美新”临沂建设取得令人瞩目的成就。

——综合实力跃上新台阶。地区生产总值年均增长9.5%，2016年突破4000亿元大关，是2011年的1.5倍，列中国城市GDP百强第45位；地方一般公共预算收入年均增长15.8%，2016年达到293.9亿元，比2011年翻了一番多；固定资产投资、社会消费品零售总额、进出口总额年均分别增长17%、12.6%和13.5%，金融机构存贷款余额年均分别增长16.3%和17%，主要经济指标增幅均居全省前列。各县区、开发区离赶超目标越来越近，兰山区列全国科学发展百强区第51位，经济技术开发区进入全国同类开发区前60强。城市知名度和美誉度大幅提升，荣获全国文明城市和国家卫生城市“两连冠”、全国双拥模范城“四连冠”，被授予国家森林城市、全国科技进步先进市和“一带一路”物流发展贡献奖等荣誉称号。

——产业结构实现新优化。三次产业比例由9.7：48.5：41.8调整到8.9：43.1：48。工业总产值年均增长10.8%，成为山东省第6个、全国第29个工业产值过万亿元的地级市；战略性新兴产业、高新技术产业产值年均分别增长13.4%和18.2%；经济技术开发区和蒙阴县、临沭县成功创建全国知名品牌示范区，数量居全国地级市首位。服务业增加值年均增长10.2%、占比稳居全省前3位，旅游消费总额占GDP比重达到15.7%。新建各类优质农产品基地108万亩，入选山东省首批知名品牌数量第一，4个品牌入围中国农产品区域公用品牌价值百强。

——城乡面貌呈现新变化。常住人口城镇化率达到56.5%、年均提高1个百分点以上。中心城区建成区人口和面积分别发展到212万人、220平方公里，临沂已成为山东省五大中心城市之一。新开通国内航线15条、国际航线2条，航空口岸获准临时开放。临枣高速、环城快速路、北疏港公路、沂沭铁路、蒙山和双岭高架路、临工大桥、西安路祊河桥、陶然路和南京路沂河桥等建成通车，鲁南高铁临沂至曲阜段、临沂机场改扩建等重点工程进展顺利。全市新增公路、铁路通车里程4622公里和173公里，“三环十五射”城市骨干路网初步形成。

——改革创新释放新动能。企业改制和“二次创业”成果明显，国有企业和规模以上集体企业、民营企业分别完成规范化改制任务的51%和57%、36%，天元、沂州集团入选“2016中国企业500强”，金锣、金升、金正大等7家企业入围“2016中国民营企业500强”。“放管服”等重点领域改革扎实推进，发展活力不断增强，各类市场主体达47.9万户，年均增长27.5%。农村集体土地确权登记颁证顺利完成，土地经营规模化率达到48%，供销社综合改革走在全国前列。新旧动能转换加快，高新技术产业产值占比提高到28.5%；新建国家级创新平台42家，中印软件园被科技部评为“国际创新园”和国际科技合作基地，河东农高区和莒南农业科技园创建成为国家级农业科技园区。

——民生保障取得新成效。各级财政民生支出年均增长14.7%，累计达到1893亿元，占财政支出比重保持在80%以上。城镇和农村居民人均可支配收入年均分别增长10.4%、11.2%。117万人实现脱贫。上学难、看病贵等群众关心的热点问题得到很大缓解。

五年来的工作，主要抓了以下六个方面：

(一)狠抓“三引一促”增强发展后劲。引导各级各部门立足职能、发挥优势，面向重点区域和优势企业引资金、引技术、引人才，促进科学发展。2012年以来，共引进项目2357个、市外资金3832亿元、“千人计划”专家等高层次人才509名、各类先进技术2100余项。累计完成固定资产投资1.4万亿元，众泰汽车、修正药业、宜华木业等一批工业项目陆续投产，天蒙景区、岱崮地貌旅游区、极地海洋世界等一批旅游项目初见成效。

(二)利用“三个工具”倒逼产业转型。全面落实供给侧结构性改革“五大任务”，综合运用环境保护、安全生产和节能减排“三个工具”倒逼产业转型升级。近两年共淘汰钢铁产能615万吨、焦化产能260万吨、非煤矿山产能960万吨，改造提升建陶生产线2.6亿平方米。制定工业转型升级“1+11”行动计划，出台支持企业“二次创业”28条政策，推动传统产业“退城进园”、走高端高质高效发展之路。五年来，累计实施工业技改项目3747个，完成投资4398亿元。国家林产工业科技示范园成为传统产业转型升级的样板区。

(三)融入“一带一路”加快商城国际化。扎实开展国际贸易省级综合改革试点，大力推进内贸与外贸、线上与线下、商贸与生产融合发展，商城国际化取得突破性进展。贸易便利化水平明显提升，“市场采购”贸易方式试点政策获批落地，综合保税区封关运行，“临满欧”等国际国内货运班列相继开行，智慧商城和临沂港运营良好，商博会入选中国十大品牌展会，临沂商城价格指数在国家级平台正式发布。国际商贸城等商贸物流重点项目有序推进，鲁南铁路物流园被列为国家亮点工程，匈牙利中欧商贸物流合作园区被认定为首个国家级境外商贸物流型经贸合作区，巴基斯坦瓜达尔港等一批“海外临沂商城”加快建设。临沂商城市场交易额年均增长21%，2016年达到3784亿元，带动全市物流总额、电子商务交易额分别完成2.7万亿元和2172亿元。

(四)实施“四级联动”统筹城乡发展。坚持规划建设一盘棋、地上地下一起建、文化产业双融合，积极推动城镇发展由外延扩张向内涵提升转变。中心城区新一轮控规实现全覆盖，乡镇总体规划修编基本完成，全市第一部实体性地方性法规《临沂市城乡规划条例》将于今年3月1日起施行。新增雨洪资源利用能力近2亿立方米，城市集中供热、管道燃气、公共供水普及率分别达到60%、79%和81%，临沂成为全国首个“镇镇通”天然气的地级市。大力推进城乡协调发展，沂水县、兰陵县被省政府批准为“50万人口以上的中等城市”试点，经济技术开发区被列入国家新型城镇化综合试点和产城融合示范区，兰山区义堂、费县探沂等小城镇被列入全国特色小镇和省级重点示范镇创建培育试点。“数字城管”延伸到社区，城乡环卫一体化在全省率先覆盖全市。

(五)开展综合整治优化生态环境。全面打响大气污染防治攻坚战，环境保护与经济发展实现互促共赢，空气综合质量近两年稳居全省第7位，国家环保部和省委、省政府对“临沂样本”给予充分肯定。深入开展水环境综合整治，增建治污项目1122个，11个国控河流断面水质全部达标，代表山东省迎接国家淮河流域核查夺得“八连冠”。加强生态修复，完成水土流失治理面积1500平方公里，扩大恢复和改善灌溉面积240万亩。大搞造林绿化，打造绿色屏障，新增造林152万亩，森林覆盖率提高到35.6%，沂蒙山国家地质公园入选世界地质公园推荐名录。推行排污权有偿使用和交易制度，较好地完成了省政府下达的节能减排约束性指标。

(六)办好惠民实事保障改善民生。持续开展全民创业行动，新增城镇就业65万人、农村劳动力转移就业104万人，城镇登记失业率控制在3.5%以内，县区“零工市场”和创业大学全部建立。不断完善社会保障体系，居民基础养老金每人每月提高到100元，城乡低保和农村“五保”对象合规费用医疗救助比例达到 70%以上，残疾人康复机构覆盖所有乡镇，257万农村人口饮水安全问题得到基本解决。大力推进教育均衡发展，新建、改扩建城镇学校122所，消化了53%的“大班额”，完成了262个农村学校的“全面改薄”项目，所有县区通过国家县域义务教育均衡发展评估认定，临沂技师学院被确定为“国家级高技能人才培训基地”，临沂大学成为山东省应用型人才培养特色名校。稳步实施县级公立医院改革，23家县级和17家城市公立医院全部取消药品加成，国医堂在乡镇卫生院普遍设立。繁荣文化事业，沂蒙革命纪念馆、临沂大剧院建成启用，多数农村建有文化大院和农家书屋，柳琴戏《沂蒙情》获“五个一”精品工程奖。积极发展体育事业，农村及城市社区健身设施覆盖率分别达到80%和90%。努力改善群众居住条件，共建设保障性住房3.9万套，完成棚户区改造15万户，改造老旧小区330万平方米、农村危房5.3万户。全面加强平安临沂建设，安全生产和社会治安形势平稳向好。通过开展安全生产“大快严”行动，扭转了安全生产的被动局面，去年较大及以上安全生产事故起数同比下降43%、死亡人数同比下降71%。加快公路安全生命防护工程建设，整治安全隐患里程6300多公里。健全四级食药安全监管体系，创建“食安山东”品牌352个。深化精神文明创建，倡树社会主义核心价值观，移风易俗广泛开展。协调发展各项事业，国防动员、人民防空、民兵预备役、双拥共建工作取得新成绩，对台、外事、侨务、老龄、气象、史志、档案、保密、统计、红十字会、防震减灾、妇女儿童、民族宗教和对口支援、扶贫协作等工作都有新的进展。

各位代表，过去五年，在各种困难较大、挑战增多的情况下，全市各级政府和广大干部群众团结一心、履职尽责，做了不懈努力。五年来，我们按照为民务实清廉的要求，扎实开展党的群众路线教育实践活动、“三严三实”专题教育和“两学一做”学习教育，作风加快转变，效能全面提升。持续加强法治政府建设，简政放权、放管结合、优化服务同步推进，市县权力事项分别精简34%和27%，468项市级行政许可和权力事项进驻新建成的政务服务中心，办理时限缩短一半以上。勇于改革创新，财税金融等17项国家级和16项省级改革试点取得良好效果。自觉接受人大法律监督、政协民主监督和群众社会监督，人大代表建议和政协委员提案全部如期办结。着力提高市民服务热线办理实效，群众诉求办结率达99%。严格执行中央八项规定及省、市委实施办法，依法加强行政监察和审计监督，严查各类违法违纪问题，各级政府廉政建设提高到一个新水平。

五年来的实践进一步启示我们：必须创新理念引领发展。主动适应经济发展新常态，践行新理念，在“转”中提档升级，在“调”中优化结构，在“创”中增添动力。必须彰显特色发挥优势。坚持一体化布局、差异化定位、特色化发展，充分发挥“山水之城、文化之邦、商贸之都、创业之地”城市品牌优势，打造新亮点、实现新突破。必须勇于担当攻坚克难。大力弘扬新时期沂蒙精神，以积极进取的“上行动力”应对宏观经济的“下行压力”，不断爬坡过坎，开创崭新局面。必须不忘初心真情为民。恪守为民之责，多办利民之事，千方百计保基本、补短板、兜底线，以实际行动赢得群众的信赖和支持。必须团结奋进真抓实干。始终保持“功成不必在我”的境界，一张蓝图绘到底，一届接着一届干，持续用力、久久为功，实现各项工作争先进位。

各位代表，五年的成绩有目共睹、来之不易。这是中央、省委省政府和市委正确领导的结果，是市人大、市政协监督支持的结果，是全市上下凝心聚力、拼搏进取的结果。在此，我代表市政府，向全市人民、各界人士、驻临人民解放军、武警官兵和中央、省属单位，向关心和支持临沂发展的国内外朋友，表示衷心的感谢！

各位代表，前进路上无坦途。我们清醒地认识到，当前发展中还存在一些困难和问题。主要是：民间投资和工业增长比较缓慢，经济下行压力依然较大；传统产业层次不高，新兴产业比重较小，企业创新能力不足，转型发展任重道远；基础设施建设相对滞后，大气、水、土壤等污染问题仍比较突出，脱贫攻坚任务艰巨，安全生产形势不容乐观，民生保障存有一些短板，群众还有不少意见；有的政府工作人员能力不足不会为、动力不足不想为、担当不足不敢为，营商环境尚需继续优化，等等。对此，我们一定高度重视，采取切实措施，尽快加以解决。

二、今后五年的主要目标和任务

未来五年，是全面建成小康社会的决胜期，是推动供给侧结构性改革取得重大突破的攻坚期，也是加快建设更高水平“大美新”临沂的关键期。我们前进道路上尽管还会遇到不少困难，但总体上机遇大于挑战。从宏观大势看，世界新一轮科技革命和产业变革蓄势待发，我国新旧动能转换正在加快，经济发展速度继续走在全球前列。从政策趋向看，中央继续实施积极的财政政策和稳健的货币政策，宏观调控更加精准有力，对新技术新产业的支持越来越大，政策红利将进一步释放。从我市情况看，经过多年发展，经济总量不断扩大，综合竞争优势持续提升，一批重大基础设施和产业项目相继投运，发展基础更加坚实。只要我们抓住机遇，发挥优势，团结实干，就一定能够把握发展主动权，开辟发展新境界。

今后五年，政府工作的总体思路是：深入贯彻党中央国务院治国理政的新理念、新举措，用心落实习近平总书记系列重要讲话特别是视察山东及临沂重要讲话精神，按照省委、省政府和市第十三次党代会部署，坚持稳中求进工作总基调，以提高发展质量和效益为中心，以供给侧结构性改革为主线，以科技创新和改革开放为动力，继续实施富民兴临“八大战略”，协调推进经济、政治、文化、社会、生态文明建设和政府自身建设，努力在全面建成小康社会进程中“走在前列”。

全市经济社会发展的主要目标是：到2021年，地区生产总值力争突破6000亿元、年均增长8%左右，地方一般公共预算收入达到450亿元、年均增长10%左右；固定资产投资、社会消费品零售总额和进出口总额年均分别增长13%、11%和8%左右，城乡居民人均可支配收入分别提高到45000元、17000元；全市经济综合实力迈入山东省第一方阵，与全省同步全面建成小康社会。

—— 建设现代产业新城。紧跟“中国制造2025”和“互联网+”产业发展新趋势，实施“战略性新兴产业倍增”计划，强化创新驱动，推进工业化信息化深度融合、服务业制造业协同配套，构建高端高质、多极支撑的现代产业新体系。到2021年，打造健康食品、冶金建材2个“三千亿级产业”，机械制造、绿色化工、高端木业3个“两千亿级产业”，新型医药、纺织服装2个“千亿级产业”，新材料、新信息、新能源汽车三大战略性新兴产业产值突破千亿元，规模以上工业企业主营业务收入达到 1.5万亿元；争取成为鲁南苏北地区现代服务业发展先行区；整建制创建国家农产品质量安全市。

—— 建设商贸物流名城。深度融入“一带一路”国家发展战略，用足用好“市场采购”贸易方式试点政策，扎实推进综保区、航空物流枢纽、国际商贸城等重点项目，着力构筑全国有影响的商品交易批发中心、物流分拨中心、电商集聚中心和“一带一路”国际贸易高地、国际会展经济高地，通过做大做强商贸物流，带动全市开放型经济更快发展。到2021年，临沂商城市场交易额突破8500亿元，全市物流总额、电子商务交易额分别达到3.5万亿元和6500亿元左右。

—— 建设生态宜居水城。按照“水城”建设总体规划，联动开发“水、岸、滩”，一体打造“堤、路、景”，贯通水系、净化水体、提升景观，形成“碧水绕城、城水相依”的滨水城市风貌。加快北城新区二期、高铁片区和西部国际商贸物流园区、南部循环经济区、东部生态城建设，推动莒南县、临港经济区与中心城区一体化发展。完善立体大交通体系，争取鲁南高铁通车，京沪高铁二通道开建，岚罗高速、新台高速和张临高速投入使用。改造国省道600公里、农村公路3000公里，临沂机场技术等级升格为4D级，力争建成全国性综合交通枢纽城市。继续推动县改市、镇改街、村改居，培育新生中小城市和特色小镇。到2021年，全市常住人口、户籍人口城镇化率分别达到63%和53%左右。

—— 建设红色文化圣地。实施红色文化资源保护、理论研究、开发利用、精品创作、宣传教育“五大工程”，提升沂蒙革命纪念馆、孟良崮战役纪念馆、省政府旧址展馆、沂蒙红嫂纪念馆、大青山胜利突围纪念馆等“沂蒙红色展馆群”，争取成为中国红色文化教育培训示范区，让“红色文化圣地”品牌享誉全国。进一步挖掘书圣、智圣、孝圣、算圣等历史文化的内涵，搞好文化古迹的保护和推介，打造传统与现代相融合的文化大市。到2021年，建成“十大红色旅游景区”和“十大文化精品园区”，全市文化产业占GDP比重达到4%以上。

—— 建设美好和谐福地。坚持民生优先，集中力量办好惠民实事，编牢织密民生保障网，增强群众幸福感。全市各级民生投入占比保持80%以上，高于全省平均水平和周边地市。着力抓就业带民富，城乡居民人均可支配收入年均增幅高于GDP、农村居民收入增幅高于城镇。进一步健全社保体系，推进基本公共服务均等化，努力让老区人民“劳有多得、学有优教、病有良医、住有宜居、出有畅行、贫有善济”，过上更加幸福美好的新生活！

三、2017年工作安排

今年是实施“十三五”规划的重要一年，也是供给侧结构性改革的深化之年，做好今年工作，意义尤为重大。综合考虑各方面因素，确定2017年全市经济社会发展的主要目标是：地区生产总值增长8%左右，地方一般公共预算收入同口径增长9%左右，固定资产投资和社会消费品零售总额均增长11%左右，进出口总额增幅高于全国全省平均水平，城镇和农村居民人均可支配收入分别增长8%、8.5%左右，城镇登记失业率控制在3.5%以内，全面完成省政府下达的节能减排降碳约束性指标。突出抓好六个方面的工作：

(一)着力振兴实体经济。正确处理“稳”和“进”的关系，在经济增速保持合理区间的前提下，下大力气推动产业转型升级，做大做强实体经济。坚持工业强市战略不动摇，依靠创新驱动，培育现代产业体系，加快新型工业化步伐。以食品、机械、冶金、纺织行业为重点，实施机器换人、产品换代、节能减排、安全增效为主要内容的“千企千项千亿”技改工程，推动传统产业提高技术和装备水平；以钢铁、建材、化工、木业行业为重点，实施以去产能、提标准、增特色为主要内容的整合重组工程，提高发展质量和效益；以高端装备、新医药、新材料、新信息、新能源行业为重点，实施以“两化融合”为主要内容的智能制造工程，培育发展新动力。实施龙头企业、中小微企业、高新技术企业创新行动，启动金融支持企业创新计划，落实好加计扣除、税收减免等优惠政策，强化军民产业融合，鼓励企业对标赶超，抓好关键核心技术研发推广，积极发展大数据经济、分享经济、创意经济、文产经济等新业态，增强企业核心竞争力。实施“5311”企业家培养计划，重点培养5名能够带领企业进入国内500强的引领型企业家，30名能够带领企业在全国同行业处于领先地位的骨干企业家，100名具有全球视野、通晓国际规则的优秀企业家，1000名具有良好发展前景的潜力企业家，示范带动全市企业管理者增强现代经营理念，提高职业素养，为企业长远发展夯实基础。适应多样化、个性化消费需求，提高生产性服务业比重，优化生活性服务业结构。规范发展互联网金融，大力引进国内外保险、证券、期货等非银行金融机构入驻临沂。培育信息服务、研发设计、节能环保、软件开发、居家养老和医疗健康服务等高附加值产业，推广个性化定制、装配式建筑等新模式，推动服务业向专业化和价值链高端延伸。围绕构建“一核两圈三带五区”旅游发展格局，集中建设一批龙头景区和精品线路，争创国家全域旅游示范市。加快发展农业“新六产”，推动标准化、规模化、品牌化生产经营，在一二三产融合发展中实现增收增效。坚持以“稳粮、优经、扩饲”为抓手，推进粮食高产田创建、“临郯苍”粮仓建设和良种培育工程，确保粮食总产稳定。执行最严格的耕地保护制度和节约集约用地制度，牢牢守住建设用地总量和强度“双控”目标，确保完成单位国内生产总值建设用地使用面积下降的年度任务。推动农村生产要素优化配置，培育新型农业经营主体。大力发展设施农业、精准农业和精深加工业，加强农村水利设施建设、运营和管理，推动农业全环节升级、全链条增值。建立农产品质量可追溯体系，抓好农药、化肥减量增效，不断增加绿色优质农产品供给。大力实施质量强市、标准引领和品牌带动战略，引导企业发扬工匠精神，制定实施质量提升计划，实施“二次创业”，培育“百年企业”。依法加强知识产权保护，严厉打击各种制假售假行为。企业、政府、媒体和社会各界要一起发力，努力把金锣、临工、金正大、鲁南制药、罗欣药业、兰陵美酒等一批工业品牌叫得更响，苍山大蒜、郯城银杏、蒙阴蜜桃、沂南黄瓜、平邑金银花、临沭柳编等一批农产品卖得更好，蒙山生态游、沂水峡谷游、竹泉乡村游、汤泉休闲游、沂南红色游和九间棚山村游等一批旅游品牌打得更远，让“产自临沂”品牌走向全国、迈向世界！

(二)深度融入“一带一路”。“一带一路”发展战略是中央从战略和全局高度作出的一项重大决策。在当前贸易保护主义抬头、对外贸易困难加大的情况下，融入“一带一路”战略，是我市发展开放型经济的重大机遇。我们要牢牢抓住这一机遇，发挥自身优势，集中力量建设“商贸物流名城”，以商城国际化引领双向开放上水平。加快建设国际商贸城，促进电子商务产业园、山东高速现代物流区域总部等重点项目早日建成运营，打造临沂商城国际贸易发展样板区。着力提升老商贸城，力争用2到3年时间基本完成传统市场改造任务。建立面向临沂商城的地产品加工专业区，抓好优势地产品出口，提高临沂商品国际市场占有率。规范发展网上商城，争取阿里巴巴、京东、苏宁云商等一批知名电商在临沂设立区域营销中心，培育一批跨境电商龙头企业。加强海外商城建设，稳步推进巴基斯坦瓜达尔港等一批在建项目，支持有条件的企业走出去共建“海外仓”和“边境仓”。规范提升公路物流，实施好鲁南铁路物流园、山东高速物流园、航空物流区和货运枢纽工程、公铁联运物流园区等项目，吸引菜鸟、京东等知名物流企业在我市设立分支机构或分拨基地。办好第八届商博会和第四届资本交易大会，打造木博会和建材展、五金展、汽车用品展等品牌展会，争取举办有一定规模的各类会展150场次以上。联手央企国企“走出去”，加强与大型国际承包工程企业的合作，扩大临沂工程物资等大宗商品出口。支持工商联用好商会平台组织民营企业参与“一带一路”建设。借鉴金正大等企业海外并购经验，鼓励临工、天元等企业和外贸经营主体开展国际产能合作，带动我市骨干企业和优势产品携手开拓国际市场。

(三)统筹推进城乡一体化发展。实行“多规合一”，加快新一轮城市总体规划编制，完成《高铁片区空间发展概念规划》《城市轨道交通近期建设规划》等重点专项规划，进一步完善现代规划体系。严格实施《临沂市城乡规划条例》，年内基本完成城市重点违法建设的治理任务。大力推进临沂至曲阜、临沂至日照鲁南高铁建设和岚罗、新台、京沪改扩建等高速公路建设，扎实做好京沪高铁二通道、张临高速和中心城区轨道交通建设前期工作，尽快完成新西外环、北京东路改造和中环北线、东线建设，不断提升普通国省道和农村公路技术等级水平，争创全国综合运输服务示范城市。着眼“生态修复”，完成海绵城市建设年度任务，新增中心城区绿地200万平方米；开展“绿满沂蒙”行动，加强湿地修复保护，抓好环城生态防护林带、退耕还林、荒山绿化和绿色通道提升工程。加快棚户区改造，推行棚改货币化补偿、存量房安置办法，确保改造棚户区3万户，货币化安置率60%以上。进一步完善园区基础设施，推动县城公共服务功能向园区延伸，提升园区综合承载能力，切实把园区建设成为产业化与城镇化联动发展的重要载体。加快推进新市镇发展，鼓励县区建成一批产业特色鲜明、创业创新互动、生态环境优美、多种功能融合的“特色小镇”。发挥水脉文脉绿脉等优势，加强特色村落保护与挖掘，抓好“美丽乡村”标准化、城乡环卫一体化、铁路沿线环境整治等工作，培育一批具有示范带动作用的“美丽乡村”。加强物业公司管理，推进小区物业服务规范化。进一步放开落户限制，全面落实流动人口居住证制度，统筹提供基本公共服务，让农业转移人口和外来务工人员“进得来、落得下、过得好”。

(四)全面深化重点领域改革。深入推进供给侧结构性改革，积极稳妥去产能，用好环境保护、安全生产、节能减排“三个工具”，推动陶瓷、化工等企业“退城进园”，坚决清理取缔“地条钢”等钢铁违法违规项目，确保完成120万吨生铁和20%的建陶产能压减任务。分类施策去库存，支持刚性和改善性住房需求，保持房地产市场平稳健康发展。千方百计去杠杆，鼓励企业盘活存量资产，实行债转股，降低负债率。严格管理降成本，认真落实清费减负政策，引导企业加强内部管理，多方挖潜增效。从制约经济社会发展的重要领域和关键环节入手补短板，努力扩大有效供给。积极推进农村土地所有权、承包权、经营权“三权分置”改革，抓好承包土地经营权和住房财产权抵押贷款试点，以及集体资产股份合作制试点。扎实开展土地二级市场试点，尽快建设产权清晰、市场定价、信息集聚、交易安全的土地二级市场。按照现代企业制度要求，加快规模以上集体企业和民营企业规范化改制，年内基本完成改革任务。进一步理顺经济园区管理体制，创新用人、薪酬等激励政策。改进预算管理，预算支出重点向供给侧结构性改革、基本公共服务和民生领域倾斜。健全综合治税体系，保障财政收入稳步增长。支持临商银行拓展经营规模、创新金融产品，完善农商行法人治理结构，鼓励村镇银行在乡镇布设网点、拓展业务，稳妥推进新型农村合作金融试点。规范PPP项目运作，推动政府向社会购买服务，吸引政策性金融机构增加贷款投放，支持通过资产证券化方式实现市场化融资。继续抓好“三引一促”，更加注重面向国际国内重点区域、战略性新兴产业和优势企业，引进一批体量大、效益高、前景好的项目，提高招引实效。深化科技创新体制改革，建立以增加知识价值为导向的分配机制，允许科研人员、医生和高职高校教师依法依规适度兼职兼薪。继续加强公立医院、科研院所法人治理结构建设，涉及单位全部落实法人自主权。加快中小学校长职级制和去行政化改革，推行教师“县管校聘”管理，不断激发教育事业发展活力。

(五)坚决打好环保治理攻坚战。持续开展“铁腕治气”，严格执行大气污染物第三时段排放标准限值，健全联防联控、联合惩戒机制，对钢铁、焦化等行业企业实施深度整治，着力加强石化、有机化工等行业挥发性有机物治理，统筹抓好城市扬尘、机动车尾气、秸秆焚烧和散煤治理，确保空气综合质量排名稳居全省前10位。全面实施“综合治水”，执行最严格的水资源管理制度，严守水资源开发利用控制、用水效率控制、水功能区限制纳污三条红线。完善“治用保”体系，率先实行“河长制”，不折不扣地完成省政府下达的城市黑臭水体治理任务。建成使用沂河河湾和袁家口子水源工程，提高中心城区用水保障水平。科学推进“重拳治土”，大力推广绿色环保养殖和病虫害绿色防治技术，有效处置各类危险废物，年底全市危废产生单位、经营单位规范化管理抽查合格率均达到90%以上，工业固废、危废无害化处置率达到100%。调整优化能源结构，支持再生能源发展。推行环境污染“第三方治理”，探索实施城市污水、生活垃圾处理收费等交易制度，加快建立生态文明建设长效机制，努力让沂蒙老区大地常绿、空气常新、土壤常净、碧水常流！

(六)千方百计提高群众幸福指数。牢固树立以人民为中心的发展理念，着力解决群众最关心、最直接、最现实的利益问题，让千万老区人民更有获得感。坚决打赢脱贫攻坚战，着力抓好产业项目实施、涉农资金整合、资产收益分配、行业社会联动、长效机制构建等工作，努力实现由帮扶重点村向区域开发转变、由扶持绝对贫困人口向兼顾相对贫困人口转变、由实施精准扶贫向兼顾防贫转变，切实提升村集体收入、公共设施水平和贫困群众满意度，稳定实现贫困人口“两不愁、三保障”目标，确保今年基本完成脱贫攻坚任务。落实保险、低保、救助等措施，统筹解决好城市贫困问题。着力推进大众创业、万众创新，加强创业孵化基地、创客空间建设，统筹做好高校毕业生、农村转移劳动力、城镇困难人员、退役军人等重点群体就业创业。健全居民养老保险参保缴费激励机制，提高居民医保财政补助标准。全面实施机关事业单位养老保险制度，做好职工基础养老金全国统筹的衔接准备。实行农村低保线和省定扶贫线“两线合一”。整合农村“五保”供养和城市“三无”人员救助制度，保障城乡特困人员基本生活。健全农村留守儿童、妇女、老人、残疾人关爱服务体系。推动公办养老机构改革，发展医养融合型养老机构。加快改善义务教育办学条件，优化城乡学校布局，基本解决“大班额”问题，完成年度“全面改薄”任务，不断提高基础教育教学质量；协调发展各类教育，加强幼儿园和幼儿教师队伍建设，落实第二批特殊教育提升计划，搞好现代职业教育学徒制试点，支持临沂大学和青岛理工大学临沂校区建设。深化医药卫生体制改革，编制实施卫生与健康事业发展相关规划，扎实开展卫生区域创建活动和全民健康保障工程；推进卫生机构标准化建设，壮大以全科医生为重点的基层卫生人才队伍；整合卫生、计生服务资源，完善疾病预防控制体系。开展“基层公共文化服务能力提升年”活动，抓好市县文化馆(艺术馆)、图书馆和街道综合文化站、社区综合文化服务中心建设；实施文物保护与旅游结合、非物质文化遗产生产性保护和革命文物征集展示工程，努力推出一批体现区域特色的精品力作；启动市县广播电视台改革和“中央厨房”工程，促进媒体深度融合。加强县级国家综合档案馆和信息化建设，开展第二轮修志。进一步健全体育公共服务体系，实现农村健身工程县区全覆盖。深入推进平安临沂建设，实施“七五”普法规划，打好三年禁毒人民战争和公安基层基础建设三年攻坚战，建成公共安全视频监控联网应用示范城市，筑牢社会治安立体化防控体系；改进信访工作，健全社会矛盾排查预警和多元化解机制；构建公共法律服务体系，保证困难群众获得及时有效的法律援助。完善风险分级管控和隐患排查治理“两大防控体系”，深化“大快严”整治行动，确保安全生产形势持续稳定好转。加强食品药品监管，守护好“舌尖上的安全”。着力防范化解企业经营、银行信贷、政府债务等风险，坚决守住不发生系统性区域性金融风险底线。健全社会信用体系，建立守信激励、失信惩戒联动机制。加强基层政权和群众自治组织建设，搞好村民自治和村务监督。抓好水利、气象、地震、人防、消防等工作，提高防灾减灾和应急救援能力。开展文明创建、双拥共建、道德模范评选、乡村移风易俗、优良家风弘扬等活动，树立社会新风尚，凝聚发展正能量。

四、关于政府自身建设

各位代表，新常态孕育新机遇，新任务呼唤新作为。站在新的历史起点上，展望未来，我们信心满怀。各级政府必须大力弘扬沂蒙精神，不忘初心、拼搏实干，切实履行好引领发展、服务群众的神圣职责。

要勇于担当。牢固树立“四个意识”，坚定中国特色社会主义道路自信、理论自信、制度自信、文化自信，始终保持对远大理想、奋斗目标的清醒认识和执着追求，坚定政府工作的政治方向。保持终身学习的自觉性，深入学习习近平总书记系列重要讲话精神，广泛学习政府工作所必需的各种新知识、新技能，努力克服本领恐慌。保持奋发有为的精神状态，盯紧目标、埋头苦干、重抓落实。保持争先创优的工作激情，自觉在全国全省大局中谋划工作，高点定位、对标赶超，推动各项事业争上游、创一流。保持攻坚克难的作风韧劲，勇于挑最重的担子、啃最硬的骨头，当改革促进派、发展实干家。完善正向激励、容错纠错机制，客观看待干部在推动发展中出现的问题，宽容干部在工作中特别是改革创新中出现的失误，坚决查处诬告陷害行为，旗帜鲜明地为担当者担当，对负责者负责。强化督政问效，更广泛地调动干部队伍干事创业的积极性，努力形成“能者上、平者让、庸者下”的鲜明导向。

要优化服务。加大“放管服”力度，动态调整行政许可事项目录清单、行政权力清单和责任清单，再削减一批行政许可等权力事项，再减少一批行政事业性收费；加强事中事后监管，实现“双随机、一公开”制度全覆盖；办好政务服务中心和“网上办事大厅”，全面推行“三集中、三到位”，提高办事效率，让群众“进一个门、办一揽子事”，打造一流的营商环境。构建“亲”“清”新型政商关系，主动为企业排忧解难，不断激发民营经济发展活力。调整重组公共服务机构，清理处置“僵尸事业单位”，整顿规范中介机构，完成行业协会商会与行政机关脱钩任务。畅通民意诉求渠道，深入听取社会各界意见建议，增强政务服务事项和市民服务热线办理实效。认真执行新闻发布制度，推动政府部门“两微一端”建设，及时回应群众关切。严格落实领导干部联系和服务群众制度，深化“结亲连心”活动，推动机关干部深入基层、服务群众，在解难题、办实事、暖民心中赢得群众支持。

要依法行政。加快建设法治政府，更加注重运用法治思维、法治手段破解矛盾、推动工作，切实做到权力授予有据、行使有规、监督有效。健全公众参与、专家论证、风险评估、合法性审查、集体讨论决定等制度，提高依法科学民主决策水平。按照减少层次、提高效率的原则，深化市县综合行政执法体制改革，规范行政执法行为，促进公正文明执法。大力推进行政复议规范化建设，完善行政调解和行政裁决制度，进一步做好行政应诉工作。继续推进政务公开，自觉接受人大法律监督、政协民主监督，加强政府内部监督，主动接受社会和舆论监督，确保政府权力在阳光下运行。

要清正廉洁。认真履行政府系统从严治党主体责任，坚持“一岗双责”，持之以恒落实中央八项规定精神和国务院“约法三章”，继续巩固不敢腐的氛围，完善不能腐的制度，构筑不想腐的堤坝。进一步改进会风文风，提倡少开会、开短会、讲短话，控制并减少各类检查评比活动，让基层腾出更多的时间搞调研、抓落实。对庸政懒政怠政行为抓早抓小、动辄必咎，着力克服不作为、乱作为现象。加强制度建设，消除权力运行灰色地带，从源头上防止权力寻租和利益输送。坚决制止一切形象工程、政绩工程，反对任何统计数字造假。多干打基础、利长远的事，努力创造经得起群众和历史检验的实绩。始终保持反腐高压态势，严肃查处以权谋私等各类违法违纪问题，坚决纠正损害群众利益的不正之风，促进干部清正、政府清廉、政治清明。

各位代表！实干成就梦想，奋斗赢得未来。让我们在市委的坚强领导下，凝心聚力、团结奋进，撸起袖子加油干、走在前列奔小康，以“大美新”临沂建设的优异成绩迎接党的十九大胜利召开！
